# Predictive Value of SOFA and qSOFA for In-Hospital Mortality in COVID-19 Patients: A Single-Center Study in Romania

**DOI:** 10.3390/jpm12060878

**Published:** 2022-05-26

**Authors:** Cosmin Citu, Ioana Mihaela Citu, Andrei Motoc, Marius Forga, Oana Maria Gorun, Florin Gorun

**Affiliations:** 1 Department of Obstetrics and Gynecology, “Victor Babes” University of Medicine and Pharmacy Timisoara, 2 Eftimie Murgu Square, 300041 Timisoara, Romania; citu.ioan@umft.ro (C.C.); forga.marius@umft.ro (M.F.); gorun.florin@umft.ro (F.G.); 2 Department of Internal Medicine I, “Victor Babes” University of Medicine and Pharmacy Timisoara, 2 Eftimie Murgu Square, 300041 Timisoara, Romania; 3 Department of Anatomy and Embryology, “Victor Babes” University of Medicine and Pharmacy Timisoara, 2 Eftimie Murgu Square, 300041 Timisoara, Romania; amotoc@umft.ro; 4 Department of Obstetrics and Gynecology, Municipal Emergency Clinical Hospital Timisoara, 1-3 Alexandru Odobescu Street, 300202 Timisoara, Romania; oanabalan@hotmail.com

**Keywords:** SOFA, qSOFA, COVID-19, prediction

## Abstract

Two years after the outbreak of the COVID-19 pandemic, the disease continues to claim victims worldwide. Assessing the disease’s severity on admission may be useful in reducing mortality among patients with COVID-19. The present study was designed to assess the prognostic value of SOFA and qSOFA scoring systems for in-hospital mortality among patients with COVID-19. The study included 133 patients with COVID-19 proven by reverse transcriptase polymerase chain reaction (RT-PCR) admitted to the Municipal Emergency Clinical Hospital of Timisoara, Romania between 1 October 2020 and 15 March 2021. Data on clinical features and laboratory findings on admission were collected from electronic medical records and used to compute SOFA and qSOFA. Mean SOFA and qSOFA values were higher in the non-survivor group compared to survivors (3.5 vs. 1 for SOFA and 2 vs. 1 for qSOFA, respectively). Receiver operating characteristic (ROC) and area under the curve (AUC) analyses were performed to determine the discrimination accuracy, both risk scores being excellent predictors of in-hospital mortality, with ROC–AUC values of 0.800 for SOFA and 0.794 for qSOFA. The regression analysis showed that for every one-point increase in SOFA score, mortality risk increased by 1.82 and for every one-point increase in qSOFA score, mortality risk increased by 5.23. In addition, patients with SOFA and qSOFA above the cut-off values have an increased risk of mortality with ORs of 7.46 and 11.3, respectively. In conclusion, SOFA and qSOFA are excellent predictors of in-hospital mortality among COVID-19 patients. These scores determined at admission could help physicians identify those patients at high risk of severe COVID-19.

## 1. Introduction

Two years after the onset of the COVID-19 (coronavirus disease 2019) pandemic, the SARS-CoV-2 (severe acute respiratory syndrome coronavirus 2) virus responsible for this disease continues to claim victims globally. According to the World Health Organization (WHO), by the end of March 2022, more than 475 million COVID-19 cases had been reported worldwide, of which more than 6 million were deaths. Meanwhile, in Romania, about 65 thousand deaths out of 2.8 million confirmed cases of COVID-19 have been reported [1,2]. Throughout this pandemic, different scores and inflammatory markers have been successfully used to predict the severity and mortality of COVID-19 patients [3,4,5]. Other scoring systems that are used in the prediction of disease severity and mortality are SOFA (Sequential Organ Failure Assessment) and qSOFA (Quick Sequential Organ Failure Assessment) [6,7]. 

SOFA was first implemented in 1996, not to predict the outcome, but to describe a sequence of complications in critically ill patients [8]. SOFA analyzes the functions of six organ systems: respiratory, cardiovascular, central nervous system, renal, coagulation, and liver. The function of each organ system is scored from 0 to 4, and the sum of these scores gives a total score between 0 and 24. A higher SOFA score is associated with an increased risk of mortality [7,9,10,11]. 

The qSOFA score compared to SOFA is easier to use because it involves analysis of only three clinical parameters: systolic blood pressure ≤ 100 mmHg, respiratory rate ≥ 22 breaths/min, and altered mental status. A score ≥2 in patients with suspected infection outside the intensive care unit (ICU) may indicate potential sepsis and has been shown to be at least as accurate as the SOFA score in predicting their mortality [10].

The COVID-19 pandemic is not yet fully understood in terms of diagnosis, treatment, and prediction of its severity due to a lack of medical resources. Therefore, this study was conducted to evaluate the prognostic value of SOFA and qSOFA scoring systems for severity and in-hospital mortality of COVID-19 patients, respectively.

## 2. Materials and Methods

### 2.1. Study Design

This is a retrospective, single-clinic study including consecutive COVID-19 positive patients hospitalized at Timisoara Municipal Emergency Hospital between 1 October 2020 and 15 March 2021. The study was approved by the Ethics Committee of the University of Medicine and Pharmacy “Victor Babes” Timisoara (No. 22726/17, November 2021).

### 2.2. Participants

The criteria for inclusion of participants were described in a previous paper evaluating the predictive value of the 4C mortality score, CURB-65, and NEWS in COVID-19 mortality among patients admitted to the Timisoara Municipal Emergency Hospital [5]. Thus, participants included in the study had met the following criteria: (1) had been admitted to the Municipal Emergency Hospital following a positive test for SARS-CoV-2 by real-time reverse transcriptase chain reaction (RT-PCR) on a nasopharyngeal swab between 1 October 2020 and 15 March 2021; (2) had complete clinical and laboratory data documented in electronic medical records; (3) were over 18 years of age. Patients under 18 years of age or with missing data were excluded.

### 2.3. Data Collection

Two trained physicians collected patient demographic, clinical, and laboratory data from the electronic medical record system. All data were collected in a worksheet created in Microsoft Office Excel. Based on the data at the time of admission, SOFA and qSOFA scores were calculated as suggested by their developers. The SOFA score was computed from several parameters: oxygenation index (blood oxygen tension [PaO2]/fraction of inspired oxygen [FiO2]), mean arterial pressure, Glasgow Coma Scale (GCS), creatinine or urine volume, bilirubin, and platelets for respiratory, circulatory, neurological, renal, hepatogenic, and coagulation systems. The qSOFA score was calculated from three clinical parameters: systolic blood pressure ≤ 100 mmHg, respiratory rate ≥ 22 breaths/min, and altered mental status.

### 2.4. Statistical Analysis

Statistical analysis was performed using R: A language and environment for statistical computing (R Foundation for Statistical Computing, Vienna, Austria.). Categorical variables were reported as absolute count and frequency (n/%). Fisher’s exact test was performed to compare categorical variables. Depending on the normality of the distribution, continuous variables were reported as median (interquartile range) or mean (±SD). To compare continuous variables, the independent samples t-test was used for normally distributed data and the Mann–Whitney test for non-parametric data. Receiver operating characteristic curve (ROC) analysis was used for a discriminant evaluation of SOFA and qSOFA score performance. Predicted mortality performance was evaluated with the area under the receiver operating characteristic curve (AUC–ROC). To determine the independent predictive value, the SOFA and qSOFA scores were integrated into a binary logistic regression analysis. A *p*-value < 0.05 was considered statistically significant.

## 3. Results

### 3.1. Baseline Characteristics

A total of 133 patients diagnosed with COVID-19 admitted to Timisoara Municipal Emergency Hospital were included in the study, the mortality rate among them being 13.5%. The baseline characteristics and comorbidities, including hypertension, dyslipidemia, cardiovascular disease, diabetes, chronic pulmonary disease (COPD), chronic kidney disease (CKD), and cancers, of the survivors and non-survivors in this study sample are listed in Table 1.

The median values (interquartile range) of the SOFA and qSOFA scores were significantly higher in the non-survivors group compared to the survivor group (1 (3) versus 3.5 (2.75) and 1 (1) versus 2 (1), respectively).

### 3.2. SOFA and qSOFA Accuracy in COVID-19 Mortality 

**Figure 1:**
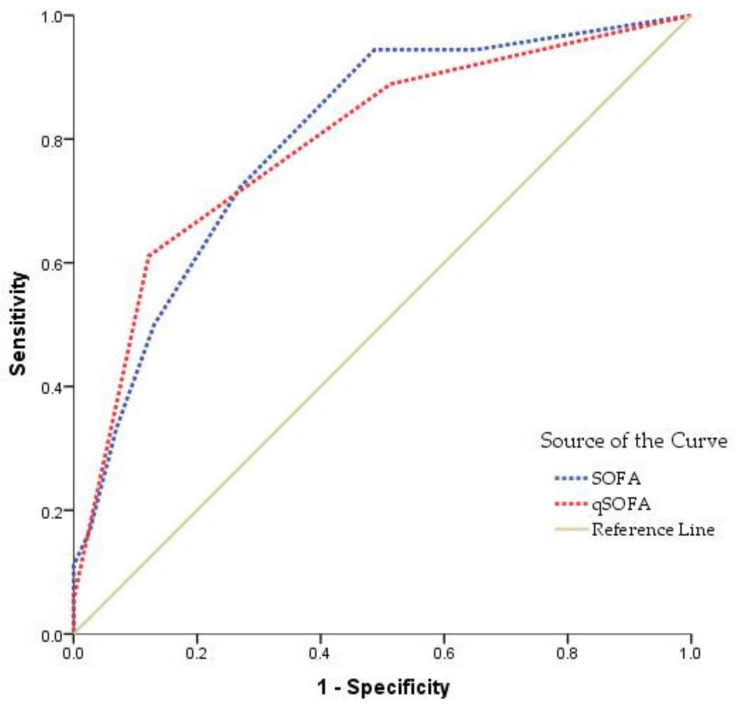
Receiver operating characteristic curve of SOFA and qSOFA in predicting mortality.

To establish the accuracy of discrimination, an AUC–ROC analysis was conducted for each individual score. ROC curves of the SOFA and qSOFA scoring systems for the prediction of in-hospital COVID-19 mortality are presented in Figure 1. Both risk scores were excellent predictors of in-hospital mortality, with AUC–ROC values above 0.6. The AUCs were 0.800 for SOFA and 0.794 for qSOFA (Table 2). 

**Figure 2:**
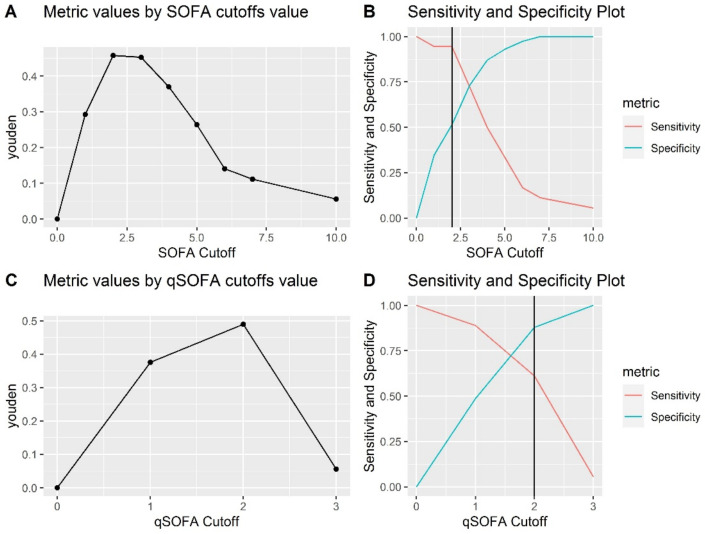
Establishment of risk score cut-off values. (**A**) Youden index according to SOFA cutoffs; (**B**) sensitivity and specificity according to SOFA cutoffs; (**C**) Youden index according to qSOFA cutoffs; (**D**) sensitivity and specificity according to qSOFA cutoffs.

Following ROC analysis, the optimal SOFA and qSOFA cutoffs in predicting in-hospital mortality by COVID-19 were determined based on the highest Youden index (Figure 2). An optimal SOFA cut-off value of 2 was assigned to predict in-hospital mortality, with a sensitivity of and a specificity of 94.4% and 51%. Similarly, an optimal qSOFA cut-off value of 2 was assigned to predict in-hospital mortality, with a sensitivity and a specificity of 61.1% and 87.8%, respectively (Table 3).

### 3.3. Regression Analysis of Mortality Risk Scores

Univariate regression analysis was conducted to assess the association between SOFA and qSOFA, respectively, and in-hospital mortality in patients with COVID-19. Both risk scores were significant predictors of mortality in the analysis with acceptable calibration (Table 4). For every one-point increase in SOFA score, mortality risk increased by 1.82, and for every one-point increase in qSOFA score, mortality risk increased by 5.23. Multivariate analysis showed that SOFA and qSOFA scores, used together, were also predictors of in-hospital mortality (Table 4).

Additionally, depending on the cut-off values of the risk scores, patients with a SOFA score greater than 2 have an increased risk of mortality compared to patients with a score below 2 (OR = 7.46, 95% CI = 1.28–43.7; *p* < 0.001). Similarly, patients with a qSOFA score above 2 showed an 11-fold higher mortality risk compared to patients with a score below 2 (OR = 11.3, 95% CI = 3.86–35.7; *p* < 0.001) (Table 5). 

## 4. Discussion

In this research, performed on a cohort of COVID-19 patients admitted to the Municipal Emergency Hospital of Timisoara, it was observed that the two scoring systems SOFA and qSOFA are powerful predictors of in-hospital mortality. Early identification of the risk of death associated with COVID-19 patients and timely and more aggressive priority treatment of these patients is particularly important in global health crises. In the absence of a specific risk-scoring system for COVID-19, numerous studies have aimed to determine the predictive value in COVID-19 mortality of validated scores in other conditions.

The SOFA score has been widely validated as a useful tool for emergency and critical-care physicians to more rapidly and accurately determine patients with increased mortality [12]. Several studies have reported SOFA scores significantly increased in non-surviving COVID-19 patients [6,13,14]

In our study, SOFA illustrated good predictive performance for in-hospital mortality on ROC curve analysis with an AUC of 0.800, being slightly higher than the ROC curve of the qSOFA score, which showed an AUC of 0.794. Other studies showing the accuracy of risk scores presented conflicting results, with AUCs between 0.679 and 0.908 [15,16]. In addition, consistent with our results, one study shows qSOFA to have less predictive value in COVID-19 mortality compared to other risk scores [5,17].

Moreover, both scores had a cut-off value in predicting mortality, established by the Youden index, of 2. SOFA had a higher sensitivity than qSOFA (94.4% vs. 61.1%), but a lower specificity (51% vs. 87.8%). Thus, both scores ≥2 may predict the severity of patients with COVID-19. Consistent with our findings, Yang et al. reported an increase of 2 or more in SOFA score, which can predict the severity of COVID-19 patients [18]. Another study showed that SOFA demonstrated significantly greater capacity compared with qSOFA in predicting in-hospital mortality among ICU patients [19]. 

Compared to other risk scores, for example, 4C mortality, CURB-65, and NEWS (The National Early Warning Score), calculated among the same patients and presented in a previous paper, SOFA had a poorer predictive value, with an AUC = 0.800 compared with 0.818 (4C mortality score), 0.861 (NEWS), and 0.801 (CURB-65) [5]. These results are consistent with other studies showing lower accuracy of the SOFA than the 4C mortality score [16]. In addition, the study by Vicka V et al. shows a lower accuracy in mortality prediction of SOFA compared to other scores such as APACHE II (Acute Physiology and Chronic Health Evaluation II) or SAPS II (Simplified Acute Physiology Score II) [16]. Regarding qSOFA, this score also had lower predictive values compared to other scores such as SIRS [20]. However, the discriminatory values of these scores presented in these studies were lower than the discriminatory values of SOFA and qSOFA determined in our study [16]. 

Various studies have identified 60 risk factors for the severity of COVID-19, of which 7 were considered of high consistency. Among the risk factors with the highest consistency as predictors of COVID-19 severity, the SOFA score was included [21]. However, the SOFA score has some complexity involving six variables, four of which are time-consuming as they come from laboratory results. Similarly, other scores such as APACHEII or the 4C mortality score involve several variables, many of which require laboratory data. Thus, the use of qSOFA is quick and practical, but there are studies suggesting that qSOFA has a low sensitivity for in-hospital mortality in patients hospitalized with suspected infection [22]. 

In terms of laboratory parameters, a study also carried out in our center, on a similar population to the one in this study, shows that coagulation parameters can be used to predict mortality. However, the accuracy of prediction of these markers is lower compared to risk scores [23].

Comorbidities (such as diabetes) are also among the predictors of COVID-19 severity [21]. However, no statistically significant difference was found in this study, except for in the cases of chronic heart disease and CKD, between the prevalence of comorbidities in the groups of survivors and the deceased. In addition, the predictive value of SOFA and qSOFA mortality scores remains high after adjusting for comorbidities and age.

This study has some limitations to be taken into account when interpreting the results. First, the study follows a retrospective design and is based on data from a single center involving a relatively small number of patients. Additionally, the sample may not have been large enough to assess the predictive performance of SOFA and qSOFA for in-hospital death as there were only 18 deaths in this cohort.

## 5. Conclusions

In conclusion, SOFA and qSOFA are excellent predictors of in-hospital mortality among COVID-19 patients, but the SOFA score had higher prognostic accuracy for in-hospital mortality than the qSOFA scale. Both scores assessed on admission could help clinicians diagnose patients at high risks of developing severe COVID-19.

## Figures and Tables

**Table 1:** Baseline characteristics of the patients.

Variable	Overall	Survivors	Non-Survivors	*p*-Value *
***Total***	**N = 133**	**N = 115/86.5%**	**N = 18/13.5%**	
*Demographics*				
*Gender (n/%)*				
Female	65/48.9%	58/50.4%	7/38.9%	0.45
Male	68/51.1%	57/49.6%	11/61.1%	
*Age*				
Median (IQR)	65 (21)	62 (20.5)	70 (15.5)	0.02
<50 years	30/22.6%	29/25.2%	1/5.6%	0.02 **
50–59 years	27/20.3%	25/21.7%	2/11.1%	
60–69 years	31/23.3%	25/21.7%	6/33.3%	
>70 years	45/33.8%	36/31.3%	9/50.0%	
*Comorbidities (n/%)*				
Hypertension	87/65.4%	72/62.6%	15/83.3%	0.11
Dyslipidemia	43/32.3%	30/26.1%	13/72.2%	<0.001
Diabetes	59/44.4%	49/42.6%	10/52.6%	0.32
Chronic cardiac disease	53/39.8%	40/34.8%	13/72.2%	<0.01
CKD	69/51.9%	55/47.8%	14/77.8%	0.02
COPD	26/19.5%	20/17.4%	6/33.3%	0.12
Cancers	15/11.3%	11/9.6%	4/22.2%	0.12
*Signs and symptoms (n/%)*				
Fever	43/32.3%	36/31.3%	7/38.9%	0.59
Cough	77/57.9%	65/56.5%	12/66.7%	0.45
Dyspnea	69/51.9%	59/51.3%	10/55.6%	0.80
Fatigue	82/61.7%	68/59.1%	14/77.8%	0.19
Gastrointestinal symptoms	52/39.1%	44/38.3%	8/44.4%	0.61
SpO2 (median (IQR))	94.0	95.0 (8.0)	92.5 (3.5)	0.10
Body temperature (median (IQR))	36.6	36.6 (0.8)	36.8 (1.9)	0.40
*Clinical course*				
Mechanical ventilation	9/6.8%	2/1.7%	7/38.9%	<0.001
Length of hospital stay	10.0	11.0 (11.0)	3.5 (4.75)	0.01
ICU admission	10/7.5%	4/3.5%	6/33.3%	<0.001
*Risk scores*				
SOFA	2 (3)	1 (3)	3.5 (2.75)	<0.001
qSOFA	1 (1)	1 (1)	2 (1)	<0.001

COPD, chronic obstructive pulmonary disease; CKD, chronic kidney disease; ICU, intensive care unit; SOFA, Sequential Organ Failure Assessment; SpO2, oxygen saturation as measured by pulse oximeter; qSOFA, Quick Sequential Organ Failure Assessment; * Statistical significance of differences between groups was determined using Fisher’s exact test (for categorical variables) and Mann–Whitney test (for continuous variables); ** Statistical significance was determined using linear-by-linear association (Mantel–Haenszel test).

**Table 2:** The AUC–ROC of SOFA and qSOFA in predicting in-hospital mortality.

Risk Score	AUC	Std. Error	*p*-Value	95% CI	
				Lower	Upper
SOFA	0.800	0.054	<0.001	0.695	0.905
qSOFA	0.794	0.060	<0.001	0.676	0.912

SOFA = Sequential Organ Failure Assessment; qSOFA = Quick Sequential Organ Failure Assessment.

**Table 3:** Sensitivities, specificities, and accuracy rates of SOFA and qSOFA for predicting in-hospital mortality.

Risk Score	Cutoff Value	Youden Index	Sensitivity	Specificity	Accuracy
SOFA	2	0.457	0.944	0.513	0.571
qSOFA	2	0.489	0.611	0.878	0.842

SOFA, Sequential Organ Failure Assessment; qSOFA, Quick Sequential Organ Failure Assessment.

**Table 4:** Univariate and multivariate logistic analysis for inpatient death of COVID-19.

Variable	OR	*p*-Value	Confidence Interval	
			Lower	Upper
*Univariate*				
SOFA	1.82	<0.001	1.35	2.46
qSOFA	5.23	<0.001	2.36	11.5
*Multivariate*				
SOFA	1.41	0.04	1.00	1.99
qSOFA	3.14	0.01	1.25	7.88

OR, Odds Ratio; SOFA, Sequential Organ Failure Assessment; qSOFA, Quick Sequential Organ Failure Assessment.

**Table 5:** In-hospital mortality odds ratio according to cut-off values of risk scores.

Variable	Unadjusted			Adjusted		
	OR	Confidence Interval	*p*-Value	aOR *	Confidence Interval	*p*-Value
SOFA > 2	7.46	1.28–43.7	<0.001	12.8	1.61–123.3	<0.001
qSOFA > 2	11.3	3.86–35.7	<0.001	23.5	5.39–146.4	<0.001

SOFA, Sequential Organ Failure Assessment; qSOFA, Quick Sequential Organ Failure Assessment; * adjusted for comorbidities and age.

## Data Availability

The datasets used and/or analyzed during the present study are available from the corresponding author on reasonable request.
